# Hyperbaric oxygen therapy for chronic antibiotic-refractory ischemic pouchitis

**DOI:** 10.1093/gastro/gov038

**Published:** 2015-08-28

**Authors:** Custon T Nyabanga, Geeta Kulkarni, Bo Shen

**Affiliations:** 1 Cleveland Clinic Lerner College of Medicine of Case Western Reserve University, Cleveland, OH, USA; 2 Department of Gastroenterology/Hepatology, Cleveland Clinic, Cleveland, OH, USA

**Keywords:** hyperbaric oxygen therapy, refractory pouchitis, ischemic pouchitis

## Abstract

Hyperbaric oxygen therapy (HBOT) has been shown to be efficacious in treating various conditions, including perianal Crohn’s disease. Here we present a case of a 59-year-old male with a history of ulcerative colitis, who underwent a total proctocolectomy and two-stage J-pouch construction. He later developed chronic antibiotic-refractory pouchitis with endoscopic features of ischemia. At the completion of HOBT—a total of 20 sessions of 100% oxygen at 2.5–3.0 atmospheres absolute for 60–90 minutes per session—a repeat pouchoscopy showed marked improvement of endoscopic mucosal inflammation. HBOT is known to increase tissue oxygenation, reduce tissue hypoxia, alter inflammatory pathways and promote tissue healing. This case demonstrated the therapeutic role of HBOT as well as the possible disease mechanism in chronic antibiotic-refractory pouchitis.

## Introduction

Hyperbaric oxygen therapy (HBOT) has been found to be safe and efficacious in the treatment of chronic wounds and inflammatory conditions, including inflammatory bowel disease (IBD) [1]. The therapy has gained favor as a first-line and/or adjuvant therapy in the treatment of carbon monoxide/cyanide poisoning, decompression sickness, air embolism, acute ischemic injuries and radiation injury [2]. More recently, its application to non-healing ulcers, skin grafts and wounds have also been described [3]. Studies have proven the safety of HBOT in the treatment of IBD, especially perianal Crohn’s disease (CD), but the efficacy of the therapy is yet to be validated through randomized, controlled trials [1]. The mechanism of the therapy is thought to be through the increase in tissue oxygenation, leading to changes in inflammatory and tissue repair pathways [2, 4]. Conditions that include tissue ischemia have been shown to improve under HBOT therapy; these findings led our group into postulating the potential benefits of HBOT in patients with chronic antibiotic-refractory pouchitis (CARP) with endoscopic features of ischemia.

## Case presentation

**Figure 1.**
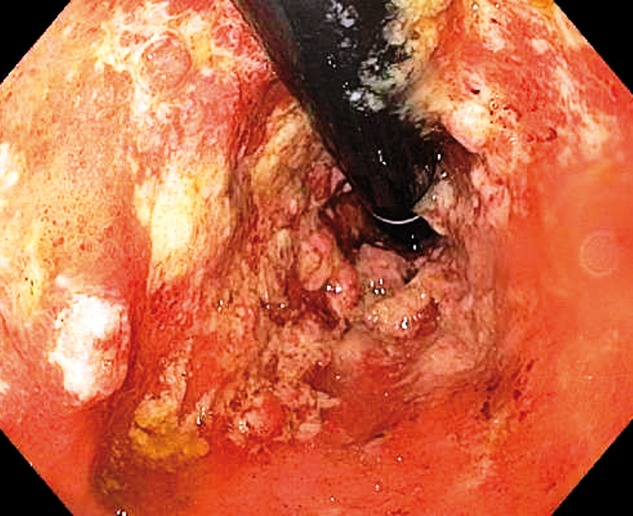
Image showing severe pouchitis with nodularity, friability and ulcers mainly in the distal pouch and the afferent limb side of the pouch body. Nodularity and ulceration were also evident in the cuff and inlet (not shown).

**Figure 2.**
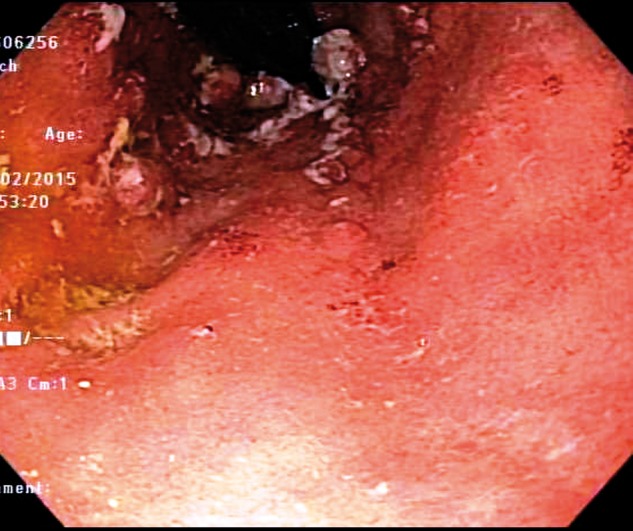
Follow-up pouchoscopy image, showing marked improvement in mucosal inflammation after hyperbaric oxygen therapy.

The patient was a 59-year-old male who presented to our Pouch Center with recalcitrant pouchitis, characterized by bowel frequency, hematochezia, urgency, lower abdominal pain and nocturnal incontinence. He was a former smoker (15-pack-years) with previous medical history of perforated diverticulitis in 2001. He was later diagnosed as having ulcerative colitis (UC) in 2007. He developed steroid-refractory UC, which necessitated surgical intervention with total proctocolectomy and a two-stage J-pouch construction in the same year. The patient remained asymptomatic following closure of the ileostomy but developed an incisional hernia 1.5 years later, which was repaired laparoscopically with mesh placement. He later developed increased bowel frequency, hematochezia, a burning sensation with bowel movements and bowel incontinence. He was diagnosed with pouchitis and treated with ciprofloxacin. In 2013, his symptoms progressively worsened, with increased lower abdominal pain and bleeding. He was treated with metronidazole and started on mesalamine suppositories. He continued to be symptomatic and additional treatment with hydrocortisone rectal foam for two weeks could not alleviate his symptoms. Pouchoscopy was performed, which showed ulcerated and nodular mucosa at the distal 10 cm of afferent limb side of the pouch body, the pattern consistent with ischemic pouchitis (Figure 1). HBOT was recommended, based on the fact that his symptoms persisted despite the therapy with anti-inflammatory drugs and antibiotics and the distribution pattern of mucosal inflammation suggestive of ischemia. The patient underwent HBOT—20 sessions of 100% oxygen at 2.5–3.0 atmospheres absolute (ATA) for 60–90 minutes each, in addition to continuing on mesalamine and hydrocortisone rectal foam. Seven weeks after this follow-up pouchoscopy revealed an approximately 65% resolution of the gross inflammation and improvement of clinical symptoms (Figure 2).

## Discussion

Pouchitis is the most common long-term complication after restorative proctocolectomy with ileal pouch-anal anastomosis. From the etiopathogenetic perspective, pouchitis can broadly be categorized into microbiome-associated, autoimmune-associated, and ischemia-associated phenotypes. CARP is one of the most challenging disorders of the ileal pouch, which can lead to pouch failure. Ischemia plays an important role in the pathogenesis of CARP [5]. The patient presented here developed inflammation of the ileal pouch, with an endoscopic pattern consistent with ischemic pouchitis, refractory to antibiotic and immunosuppressive therapies. We recommended HBOT as an adjuvant treatment for this ischemia-type CARP.

HBOT has been successful in treating IBD, particularly CD perianal disease, myocutaneous flap repairs and chronic wounds [1, 3]. The patient described here developed CARP with features of ischemia and our attempt on HBOT proved beneficial. Increased tissue perfusion with oxygen under HBOT conditions may improve tissue healing and alter the inflammatory pathways through cytokine inhibition and promotion of growth factor release and angiogenesis [4]. In this case, we demonstrated that HBOT aided mucosal healing. This observation suggests a novel and potential adjuvant therapy for individuals with CARP. On the other hand, the beneficial effect of HBOT suggests the role of compromised tissue oxygenation in the pathogenesis of chronic pouchitis.
